# Acute effects of inspiratory muscle warm-up on muscle oxygenation, perceived exertion and prefrontal cortical activation

**DOI:** 10.1371/journal.pone.0325228

**Published:** 2025-06-10

**Authors:** Xinyu Dai, Jihong Yan, Xuecui Bi

**Affiliations:** 1 Department of Physical Education, Xiamen University of Technology, Xiamen, Fujian, China; 2 Physical Education College, Jimei University, Xiamen, Fujian, China; 3 Institute of Physical Education and Training, Capital University of Physical Education and Sports, Beijing, China; Qassim University, SAUDI ARABIA

## Abstract

**Objectives:**

As rowing training loads intensify, the resulting high subjective exertion may force athletes to expand excessive neural impulses and cognitive resources. Given the finite nature of neural capacity, prolonged exposure to such demands could impair information processing, movement stability, and even causing non-contact injury. Therefore, this study aimed to investigate the effects of respiratory muscle warm-up intervention on prefrontal cortex and muscle oxygenation dynamics in athletes, thereby exploring its potential to mitigate cognitive-motor strain under rowing training conditions.

**Methods:**

A total of 54 participants were recruited for the study, with an average age of 21.35 years. They were randomly assigned to one of three groups: Inspiratory muscles warm-up (IMW), placebo, or blank control. A portable muscle oximeter (Moxy, USA) was employed to monitor the muscle oxygen saturation level of the subjects throughout the training period. The degree of activation of the prefrontal cortex (PFC) was quantified by means of an oxygenation monitoring system (OctaMon). Concurrently, the CR10 scale was utilized to assess perceived exertion.

**Results:**

Following the intervention, a significant difference in CR10 was observed between the various groups. A series of multiple comparisons demonstrated that the CR10 in the IMW group exhibited a significantly lower value than that observed in the placebo and control groups (*p* < 0.001). Once the baseline values from the pretest had been accounted for, a significant difference in oxygen saturation was observed between the different groups in the bilateral PFC. Post hoc analysis demonstrated significantly decreased HbO₂ in both the IMW (*p* = 0.019) and placebo (*p* = 0.035) groups relative to blank controls. Following the IMW intervention, the muscle oxygen saturation levels of the biceps brachii and vastus medialis in the subjects were significantly higher than those observed prior to training (*p* < 0.05). However, no significant difference was noted between the two tests in the control group, indicating that the IMW intervention can mitigate the decline in muscle oxygenation during training, alleviate discomfort in the lungs and motor muscles, and regulate subjective load.

**Conclusion:**

The implementation of IMW intervention has the potential to mitigate the subjective burden experienced by athletes, thereby reducing discomfort during training. The combination of PFC activation level and muscle oxygen saturation index provides a superior explanation of the results. Nevertheless, further research is required to ascertain whether IMW can have a sustained positive impact.

## 1. Introduction

The rising absolute velocity in modern competitive rowing and evolving pacing strategies have imposed new physiological demands on athletes. Elevated subjective load may compromise training efficacy, prompting coaches to adopt load-regulation strategies.. However, adjusting training plans by reducing or interrupting session intensity risks disrupting planned progression. Prolonged implementation of such measures may lead to insufficient chronic loads, potentially impairing athletes’ fitness development and competitive performance.

Conversely, without targeted intervention or persistent intensity escalation using motivational strategies to attain desired outcomes, the athlete’s subjective load remains excessive. Their available neural resources are finite. Sustaining elevated subjective loads long-term may demand increased neural impulses and cognitive resources to recruit target muscle groups for fulfilling training demands. This may result in an increased mental workload, which may, in turn, lead to a reduction in the information processing ability and movement stability of athletes in the later stages of training [1,2]. This is also a significant risk factor for non-contact sports injuries. Schlichta et al. demonstrated that elevated cognitive loads – which induce mental fatigue – and emotion suppression conditions may significantly impair high-intensity endurance exercise performance in young adult populations [3].In order to guarantee the implementation of the training plan and maintain a specific level of chronic load, it is not a practical approach to frequently reduce the external load as a method of load regulation. Furthermore, compared to upright running posture, seated training modalities such as cycling and rowing may compromise respiratory mechanics by reducing abdominal cavity volume and restricting thoracic excursion. This constrained posture limits diaphragmatic excursion during both contraction and relaxation phases. Prolonged maintenance of this position may consequently lead to diaphragmatic dysfunction, resulting in compensatory overactivation of accessory respiratory muscles (particularly the intercostals) and ultimately contributing to inspiratory muscle fatigue. Such respiratory limitations may adversely affect the quality and efficiency of training in rowing athletes. Targeted inspiratory muscle training may address these limitations by enhancing neuromuscular activation in diaphragmatic and accessory respiratory muscles, improving ventilatory efficiency during constrained postures. This adaptation reduces perceived respiratory effort through optimized motor unit recruitment while promoting blood redistribution to distal limbs. By attenuating respiratory muscle vasoconstriction, IMW may enhance limb perfusion, supporting oxygen delivery during endurance efforts.

To overcome this problem, the study devised a highly feasible respiratory muscle training intervention as a means of regulating the training process. This intervention was designed to reduce the subjective training load while providing a method for regulating total load and optimising training efficiency.

## 2. Methods

### 2.1. Participants

This study was conducted in accordance with the Declaration of Helsinki and approve-d by the Ethics Committee of Capital university of physical education and sports (No. 2024A183, approval date: 11 November 2024). All of the patients and healthy volunteers gave informed consent to participate in this study and all of the patients had given written informed consent for the exercise intervention.

We estimated simple size (n) using a power analysis with the following parameters in G*power. We set effect size = 0.40; alpha err prob = 0.05; power (1—beta err prob) = 0.70; number of groups = 3; The resulting sample size (n) was 54. Finally, 54 trained male rowers were recruited on 15 November 2024 to participate in the study. All the participants signed the informed consent before the experiment and participated in the study voluntarily and actively. All subjects were healthy without cardiopulmonary and metabolic diseases, and major sports injuries occurred within half a year, none of the athletes had prior exposure to structured inspiratory muscle training or participated in respiratory-focused conditioning programs before the investigation..

### 2.2. Experimental design

Participants were assigned to one of three groups: IMW, placebo, or blank control. Interventions were conducted before formal rowing training.

All athletes underwent baseline testing to ascertain consistency of 2000 m ergometer performance, oxyhemoglobin (HbO_2_) levels and other relevant indicators. Because fNIRS technique is subject to the changes of skull and brain tissue thickness in different subjects, the resting-state oxygen saturation in the prefrontal lobe was first tested in the pilot experiment. Then, according to the training requirements, the individual ergometer resistance and segment time standards of different athletes were determined to confirm the feasibility of exercise testing program. In the first formal test, all subjects carried out general preparation activities and completed the 30 min ergometer test. The training intensity was U1, and the resistance of the ergometer and the 500-meter interval time were set according to the individual competitive ability and state. Muscle oxygen saturation, perceived exertion and prefrontal lobe HbO_2_ were continuously collected during the test.

Before the second test, the control group still only carried out the routine preparation activities before rowing training, and the IMW group and the placebo group added different intensity of inspiratory muscle preparation activities. Perceived exertion during training was used as the evaluation index of subjective load. At the same time, in order to explore the possible mechanism of different interventions on subjective load, the indexes such as muscle oxygen and HbO_2_ in the prefrontal lobe of the first test were used as baseline values and included as covariates in the covariance analysis to explore the characteristics and possible reasons for the changes of indexes after the intervention.

### 2.3. Interventions

The application of moderate IMW intervention has been demonstrated to enhance the functionality of inspiratory muscles, augment the ventilation and gas exchange capacity of the lungs, and facilitate the distribution of blood to the motor muscles, while simultaneously avoiding the onset of significant respiratory muscle fatigue. Therefore, the IMW group formulated the IMW intervention program based on previous research results [4,5] and training conditions. Previous studies have found that 40% of maximum inspiratory pressure (MIP) [6] can improve the function of inspiratory muscles without causing obvious respiratory muscle fatigue and affecting the normal performance of the following training. Therefore, we first measured the MIP of all subjects with the help of Power Breath-k5, which was used as the basis for personalized setting of IMW training intensity; In the formal testing phase, IMW intervention was carried out with the help of an inspiratory muscle trainer (Power breathe plus, UK). Considering the inspiratory pressure intensity, training arrangement and the discomfort of athletes wearing the instrument, the final IMW intervention program intensity was determined as 40% MIP, and the training program consisted of two groups with 30 breaths each. The intergroup interval was set to 2 min(Table 1).The subjects were instructed to use the jaw fixed training device, and to inhale from the residual capacity until the chest could not be further expanded. Each breath was relatively soft, and the breathing time was moderately extended.

**Table 1:** IMW intervention plan design.

Groups	intervention	
**Routine warm-up**	**Inspiratory muscles warm-up**
**IMW**	5 min (e.g., pedal the cycle ergometer)	IMW(40%MIP,30 × 2)
**Placebo**		IMW(10%MIP,30 × 2)
**Blank control**		–

* Note: MIP = Maximum inspiratory pressure, IMW= Inspiratory muscles warm-up.

### 2.4. Measurements

**Fig 1:**
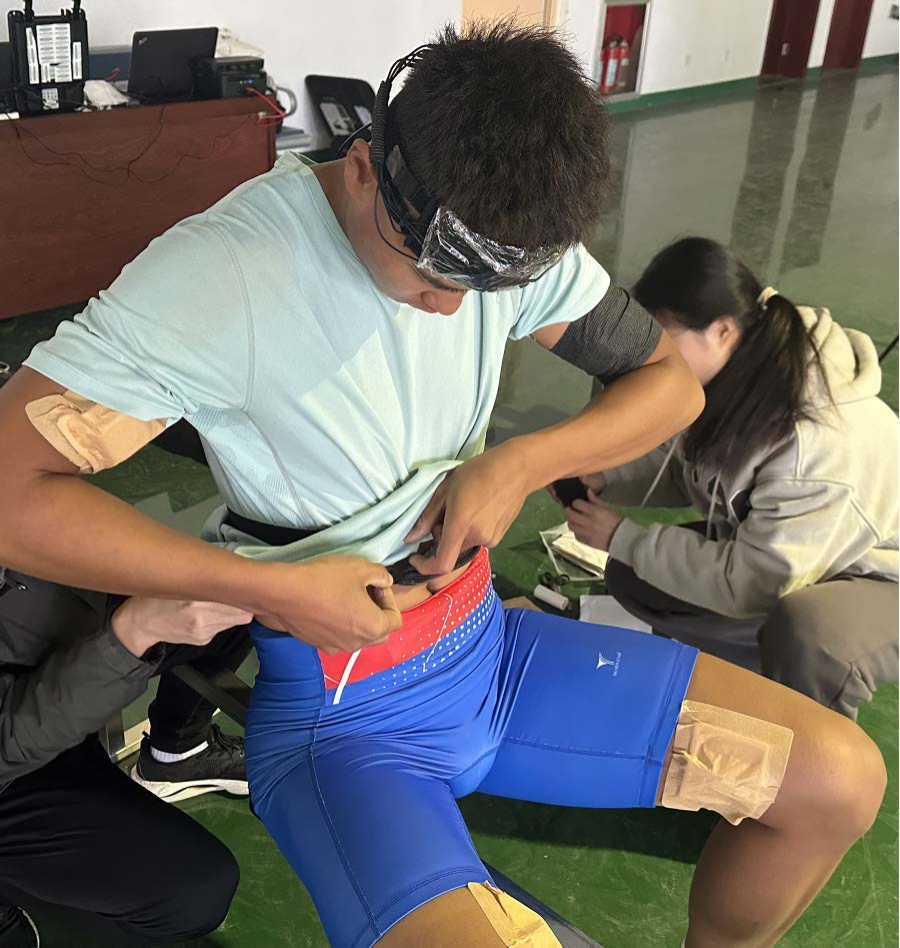
Illustration of the fNIRS device worn.

The portable muscle oxygen meter (Moxy Monitor, Fortiori Design LLC, USA) was used to continuously monitor the changes of oxygen content in vastus medialis, erector spinae and biceps brachii muscles during training. Before wearing the sensor, the muscle oxygen monitor was placed in a light shield, and then fixed on the target muscles with medical non-woven cloth. The probe was sealed with transparent tape to prevent sweat immersion, and the probe was fixed with black bandage to prevent light interference. The computer was connected by Bluetooth transmission, and the frequency of real-time sampling was set to 10 Hz. After the sensor was fixed, the formal test was started after stable changes in muscle oxygen saturation(SmO_2_) parameters were observed. Fig 1 visually shows where the subjects wore the portable fNIRS device.

HbO_2_ and Deoxyhemoglobin (HHb) were obtained according to the difference of optical density at 750nm and 850nm on the optical absorption characteristic curve to characterize the oxygen metabolism in muscle tissue. The changes of SmO_2_ during exercise and recovery period were monitored in real time from the quiet state, and the average value of every 10s during exercise and recovery period was taken as the value for analysis.

OctaMon (Artinis Medical Systems, Netherlands) monitoring system was used to collect hemodynamic parameters of the prefrontal lobe during training in athletes. This system utilizes NIRS technology to measure changes in total blood flow and blood oxygenation. It was composed of acquisition software (Oxysoft 3.2.70) and hardware, including laptop computer, sensor, battery box, etc. The subject’s forehead was fixed with a headband before testing, the width of the band was adjusted to fit the head, and the forehead hair was arranged to prevent occlusion of the sensor. In the resting state PFC activation test, the athletes closed their eyes, remained relaxed, and sat for 5 minutes before wearing the fNIRS device to continuously monitor the prefrontal hemodynamic parameters in the resting state. In the formal test, the subject first completed the preparation activity, after which the device was worn for training, and the data were recorded and saved.

**Fig 2:**
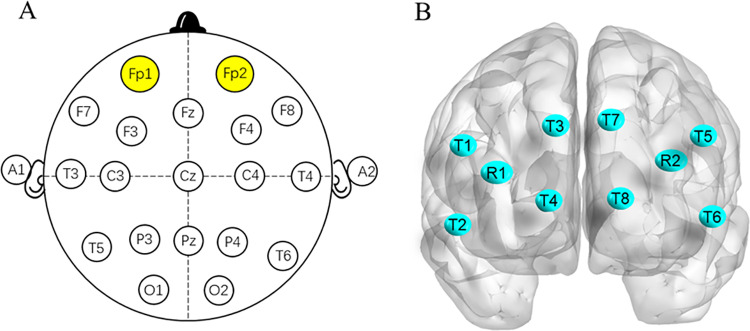
Placement on the forehead. R: receiver, T: transmitter.

fNIRS contains 8 light emitting diode light sources and 2 photodiode detectors, and the distance between the light source and the detector is about 30 mm. It weighs 200 g and does not interfere with the normal conduct of training. Two groups (4 transmit 1 receive ×2) hardware devices were used in the experiment, which were placed at electrode positions Fp1 and Fp2 of the international 10–20 system, forming a total of eight channels T1-T8 (Fig 2). After determining the coordinates of each transmitting and receiving electrode (Table 2), the eight channels were further divided into two regions of interests (ROIs) using the anatomical calibration system, namely the right prefrontal cortex (R-PFC: Channels T1, T2, T3, T4) and left prefrontal cortex (L-PFC: channels T5, T6, T7, T8).

**Table 2:** fNIRS equipment coordinates.

	x	y	z
**Receiver1**	33	61	20
**Receiver2**	−35	57	22
**Transmitter1**	49	45	32
**Transmitter2**	49	53	8
**Transmitter3**	17	69	32
**Transmitter4**	18	78	10
**Transmitter5**	−15	67	32
**Transmitter6**	−17	77	10
**Transmitter 7**	−49	37	36
**Transmitter 8**	−57	45	8

The data of the left and right four channels were averaged to represent the change level of unilateral PFC. If there was no significant difference in the level of HbO_2_ index in the left and right prefrontal lobes, the mean value of HbO_2_ index in the bilateral prefrontal lobes was presented in the results as an index for comparison before and after intervention in different groups.

The Borg Category-Ratio (CR10) scale was implemented to quantify subjective physical perception, with scale anchors explicitly defined as 0 (no exertion at all) and 10 (maximal imaginable exertion) following Borg’s perceptual range standardization.. The scale was presented immediately after training, and the subjects were asked about their immediate physical sensation. In order to avoid measurement interference in athletes’ reports in the experiment, after being familiar with the scale and referring to the suggestions of Borg E for testing. During experimental trials, only the validated scale interface was presented without additional verbal cues to prevent reporting bias, thereby maintaining ecological validity of the perceptual data collection.

### 2.5. Statistical analyses

First, the age of the subjects was filled in the Oxysoft system and the differential pathlength factor(DPF) was calculated by formula to reduce the influence of the optical properties of different individual tissues. The calculation formula is shown in Eq 1:


$$DPF=4.99+0.067\times {Age}^{0.814}$$ (1)

Oxysoft software was used to collect blood flow dynamics data from 54 subjects’ prefrontal cortex at a sampling frequency of 10 Hz. The optical density was converted into relative changes in oxyhemoglobin and deoxyhemoglobin concentrations using the modified Beer-Lambert law. In this part of the data, the focus is on the concentration of HbO_2_ in the prefrontal cortex, as previous studies have shown that compared to HHb signals, HbO_2_ signals are more sensitive to changes in cerebral blood flow.

The original data signal was converted to Optical density (OD) by the hmrlntensity2OD function. Then, in order to reduce the artifacts caused by the subject’s head motion, the PCA method was used to correct the motion artifacts of the optical density data. Subsequently, the enPCAFilter function was used for bandpass filtering (high-pass: 0.01 Hz; Low pass: 0.80 Hz) to remove noise and irrelevant physiological signals, convert the filtered optical density to HbO_2_ and HHb concentrations again by the hmrOD2Conc function, and finally calculate the average hemodynamic response function (HRF) using the BlockAvg function. The specific value of the analytical index HbO_2_ can be obtained, and the resting state index value is compared with the mean value of the whole training process of the ergometer, that is, the changes of HbO_2_ level(△HbO_2_) in the prefrontal lobe under different applied factors can be obtained.

SPSS was used for univariate statistical analysis. Before analysis, data normality test, homogeneity of variance test and baseline value comparison were performed, and the test results were expressed as mean ± standard deviation (*M* ± *SD*). Residual normality was verified using the Shapiro-Wilk test (W = 0.98, *p* = 0.37), confirming the data met parametric test assumptions. Paired sample t test was performed in each group. The baseline disparity was analyzed by Analysis of covariance (ANCOVA) and post hoc pairwise comparisons among the IMW group, the placebo group, and the control group (α = 0.05). The unrelated variables were strictly controlled during the training process. All subjects completed the closed training in the training center and the training plan formulated by the head coach. In case of discrepancies between the training plan and the actual completion, the actual completion of the training shall prevail. Each time, data were collected through the team heart rate monitoring system (Polar team pro, Finland), rowing ergometer (Concept II D, USA), paddle frequency meter (Speed coach, USA), CR10 scale, etc.

## 3. Results

### 3.1. Perceived exertion

First, the homogeneity test was conducted, and the results showed that there was no interaction effect between the group and the baseline value before the intervention (Table 3), which met the homogeneity assumption of the covariance analysis(*p* = 0.740).

**Table 3:** Homogeneity test of CR10.

	III Sums of Squares	*df*	mean square	*F*	P-value*
**model**	10.135	5	2.027	7.613	<0.001
**Intercept**	0.018	1	0.018	0.067	0.798
**groups**	0.106	2	0.053	0.199	0.821
**baseline**	3.479	1	3.479	13.065	0.001
**groups×baseline**	0.162	2	0.801	0.305	0.740
**error**	6.124	23	0.266		
**total**	1558.750	29			
**Corrected total**	16.259	28		*R*^*2*^ = 0.623	

**Fig 3:**
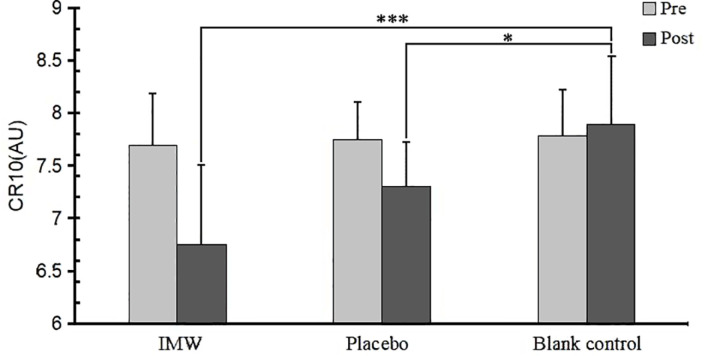
Differences in CR10 scores between different groups after intervention. ***Significantly different from pre test(*p* <0.001). * Significantly different from pre test(*p* <0.05).

After controlling the baseline value of the pretest, the difference in CR10 between different groups reached a significant level (Table 4). Multiple comparisons after ANOVA (Table 5, Fig 3) showed that the CR10 score of the IMW group was significantly lower than that of the placebo and control groups (*p* < 0.001), while the placebo group CR10 was significantly lower than the control group (*p* = 0.022).

**Table 4:** Analysis of variance of CR10.

	III Sums of Squares	*df*	mean square	*F*	P-value*
**model**	9.973	3	3.324	13.221	<0.001
**intercept**	0.012	1	0.12	0.47	0.830
**groups**	3.828	1	3.828	15.224	<0.001
**baseline**	5.384	2	2.692	10.706	<0.001
**groups×baseline**	6.286	25	0.251		
**error**	1558.750	29			
**total**	16.259	28		*R*^*2*^ = 0.613	

**Table 5:** Multiple comparisons after ANOVA(CR10).

Groups		MD	SE	P-value*	LCI	UCI
**IMW**	**Placebo**	−0.505	0.225	0.033	−0.968	−0.043
	**Blank control**	−1.069	0.231	<0.001	−1.545	−0.593
**Placebo**	**IMW**	0.505	0.225	0.033	0.043	0.968
	**Blank control**	−0.564	0.230	0.022	−1.039	−0.089

* Note: MD= mean difference, SE= standard error, CI= confidence interval

### 3.2. Brain activation

The changes of HbO_2_ concentration in each channel of the left and right prefrontal lobe of the subjects were calculated, and then the values of each channel of the subjects in each group were averaged, and combined with covariance analysis, the differences of the mean HbO_2_ concentration of the three groups after the experiment were obtained.

**Fig 4:**
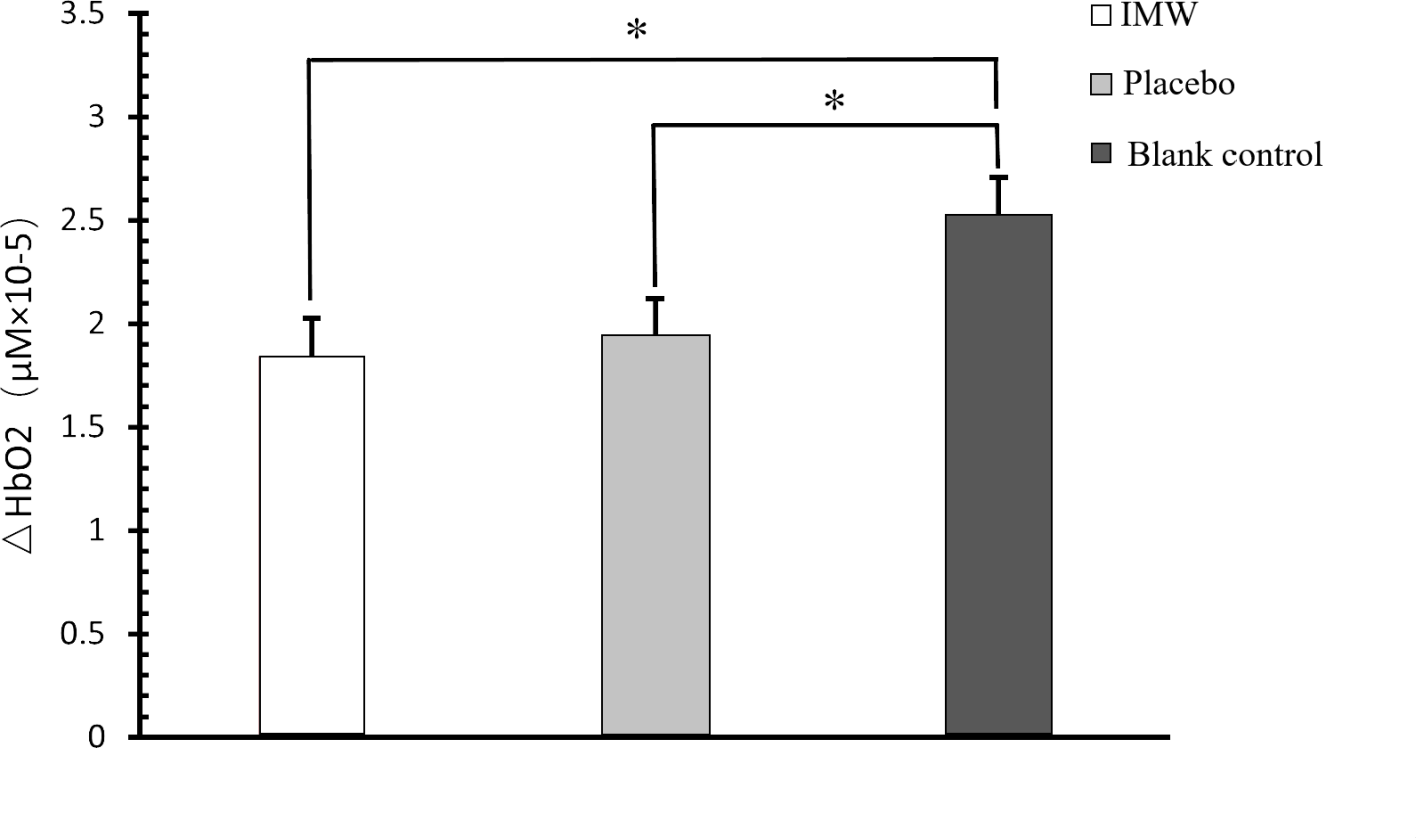
Differences in △HbO_2_ in the prefrontal cortex after intervention. *Significantly different from the other group.

There was no interaction effect between independent variables and covariates, which met the requirements of covariance analysis. Further analysis was performed again by removing the interaction term, and the model was selected to be full factor. After controlling the baseline value of the pretest, the differences in cerebral oxygenation indexes of bilateral PFC between different groups reached a significant level (Table 6, Fig 4). Post hoc analysis demonstrated significantly decreased HbO₂ in both the IMW (*p* = 0.019) and placebo (*p* = 0.035) groups relative to blank controls.

**Table 6:** Analysis of variance of HbO_2_ in bilateral PFC.

	III Sums of Squares	*df*	mean square	*F*	P-value*
**model**	47.906	3	15.969	64.328	<0.001
**intercept**	23.747	1	23.747	95.661	<0.001
**groups**	34.523	1	34.523	139.072	<0.001
**baseline**	1.885	2	0.943	3.798	0.040
**groups×baseline**	4.965	20	0.248		
**error**	159.073	24			

### 3.3. Muscle oxygen saturation

**Fig 5:**
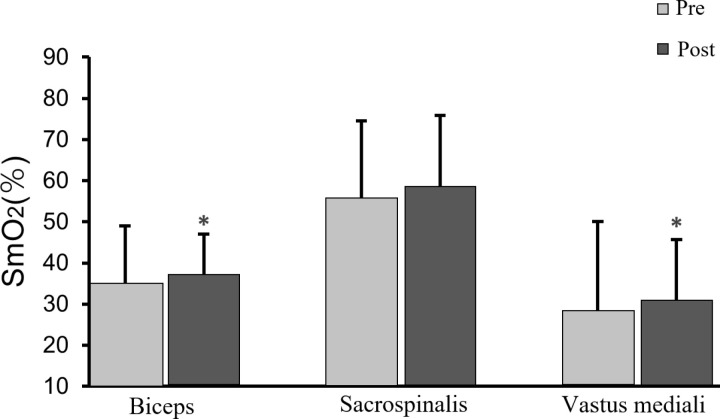
Changes of muscle oxygen saturation. *Significantly different from pre test.

Table 7 summarizes the changes of SmO_2_ level of subjects after intervention. The results of the vastus medialis and biceps brachii muscle in the IMW group were significantly higher than the pre-test (*p* < 0.05), while the changes in SmO_2_ of sacrospinalis were not significant. The control group did not produce significant changes before and after the test (Fig 5), suggesting that IMW reduces the magnitude of the decrease in the oxygenation level of the muscle during the ergometer training of athletes.

**Table 7:** The value of the SmO_2_ index in the ergometer test(*mean*±*SD*).

time	IMW(n = 18)			Blank control(n = 18)		
Biceps	Sacrospinalis	Vastus medialis	Biceps	Sacrospinalis	Vastus medialis
**SmO**_**2Pre**_	35.08 ± 13.91	57.66 ± 18.79	28.44 ± 21.65	35.33 ± 14.98	53.06 ± 17.88	34.89 ± 22.47
**SmO**_**2Post**_	37.09 ± 9.94*	58.51 ± 17.26	31.01 ± 14.69*	34.59 ± 12.33	52.84 ± 15.76	34.02 ± 15.34

* Note: SmO_2Pre_=Mean value of muscle oxygen saturation was measured before operation; SmO_2Post_=Mean value of muscle oxygen saturation after intervention;

* Significantly different from pre test.

## 4. Discussion

The essence of movement is the efficient innervation of muscles by the nervous system, and to complete training, neurons require energy substrates to generate action potentials. Due to the neurovascular coupling mechanism, when a task requires information processing in a specific brain area, local neurons are activated, which will induce the dilation of peripheral blood vessels. This will lead to a rapid increase in local cerebral blood flow, and glucose, oxygen, and other substances are delivered to metabolically active neurons. Thus, changes in HbO_2_ and HHb concentrations can indirectly reflect the functional state of the brain, while there is evidence for a linear relationship between hemodynamics and neural activity [7],It can be assumed that functional brain activation must be characterized by an increase in local blood flow or a change in local oxygenation status.

Furthermore, training represents a significant source of stimulation, resulting in an elevation of the athlete’s heart rate, cardiac output, and respiratory rate. This leads to an increase in blood flow, delivering oxygen to the skeletal muscles and brain areas, followed by tissue oxidation. Monitoring the changes of these indicators is beneficial to analyze the characteristics of athletes’ brain activity. Under the specific stimulation of rowing training, the stress state of the subjects was accompanied by the increase of local neuronal activity, and the overcompensation of blood flow in brain regions, indicating the activation of specific brain regions. In general, moderate activation improves cognitive function and makes technical movements more efficient. Nevertheless, the endurance training of rowing is of considerable duration, particularly given the high intensity of the ergometer training and the potential distance covered in a single session, which can exceed 20 km. In order to maintain high power and stability of technical movements, athletes’ brain neurons continue to be activated to complete a series of periodic technical movements such as drive and recovery. Long-term neuronal activity is easy to cause central fatigue, and then the function of the central nervous system is limited [8],will significantly affect the quality of training. To address these limitations, this study conducted a comprehensive assessment using standardized instruments based on the IMW intervention, providing a practical training reference for rowers and sports medicine practitioners.

### 4.1. Effect of IMW intervention on muscle oxygen saturation

In high-load endurance training, tissue hypoxia is one of the important causes of exercise-induced fatigue and post-exercise fatigue accumulation. Appropriate intervention can help to eliminate fatigue, improve the quality of training, and promote the recovery of athletes’ various organ systems. During the training and competition of rowing events, it is often accompanied by the exhaustion of athletes’ physical ability. The high intensity of the training schedule resulted in limited time for recovery between sessions. Especially in the training of ergometer, athletes continue to carry out periodic special movements such as drive and recovery, and the huge force when drive is easy to cause muscle micro-damage and fatigue accumulation. In the present study, there was an increase in muscle oxygen saturation in the vastus medialis and biceps brachii muscles of the subjects, indicating that IMW effectively suppressed the respiratory muscle metabolic reflex, allowing adequate perfusion of oxygenated arterial blood flow to the extremities.

Three muscles were selected for SmO_2_ testing to reflect the effectiveness of the intervention. On the one hand, in terms of rowing, vastus medialis muscle, erector spinae muscle and biceps brachii muscle are relatively important muscle groups involved in the drive phase. The study of Tomiak T [9] found that the discharge level of vastus medialis muscle was directly proportional to the speed of rowing, so our study selected vastus medialis muscle as the main muscle group for testing. The erector spinae and biceps brachii were selected as the second and third muscle groups to be tested because the RMS of biceps brachii was significantly higher when training on the dynamometer than on the water. Bazzucchi I [10] found that the biceps brachii muscles were significantly activated during the 1000m full force rowing on ergometer; On the other hand, due to the small distance between some muscles, the problem of electrode movement may occur when multiple muscle groups are monitored at the same time during training. Simultaneously, for the muscle groups of the lower limbs, the rectus femoris muscle can easily slip during contraction and can also be affected by the sartorius muscle, resulting in inaccurate signals. In conclusion, the muscle groups selected in the study can effectively reflect the characteristics of rowing specific training and support the results of the study.

During intense exercise, oxygenated hemoglobin releases oxygen to meet the needs of the body’s energy metabolism, so the level of exercise muscle SmO_2_ is reduced. Within a certain range, SmO_2_ is approximately negatively correlated with the intensity of training. In general, at the beginning of training, the muscles consume more oxygen than the oxygen supply at rest, so SmO_2_ will produce a sharp decline, and the slope of this relationship will stabilize after the body continues to adjust the oxygen supply level of the working muscles. The difference is that under the same external load, the SmO_2_ in the IMW intervention group was higher than that in the control group during training. This means that oxygen saturation in the femoral medialis and biceps decreased less in the experimental group, suggesting that IMW can improve inspiratory muscle function, inhibit the respiratory metabolic reflex and increase muscle oxygen content in the exercising muscle. Meanwhile, the subjective perception level during training is also lower than in the control group. This indicates that 40% MIP intensity acute IMW intervention designed in this study can also inhibit the afferent feedback of respiratory muscle, reduce the discomfort of skeletal muscle and cardiopulmonary system, and achieve the purpose of improving training efficiency and quality.

However, the study of Ohya T [11] does not support the positive effect of IMW on athlete performance. He designed 10 5-second intermittent bicycle exercise tests and found that, possibly due to the high intensity, short duration and relatively sufficient interval of training, the participants did not feel dyspnea during the two tests. The relevant physiological indexes of athletes cannot be changed by the activation level of respiratory muscles, so the intervention of IMW does not significantly improve the performance of the subjects. In this regard, Johnson [12] suggested that when the relative intensity of exercise exceeds 85%VO_2max_, the degree of diaphragm fatigue and the likelihood of respiratory muscle inhibition will increase, and the improvement of various indicators of subjects may reach the statistical threshold. Romer LM found that at an intensity of at least 80% ~ 85%VO_2max_, exercise to exhaustion can lead to a 15% ~ 30% reduction in trans-diaphragm pressure [13], which will limit respiratory efficiency and effective ventilation. In contrast, short, high-intensity exercise tests did not significantly affect transdiaphragmatic pressure, partially confirming Ohya T’s findings. Arend M [14] also conducted an experimental study on rowers, and included subjects who took two sub-maximum intensity rowing tests, but the results found that the only index that showed a difference between the two tests was respiratory rate. After the intervention of IMW, the subjects’ heart rate and ventilation showed a trend of decrease and increase respectively. Despite some changes in breathing parameters, the authors argue that training with 40%MIP is not recommended to warm up if the average intensity of the race is at a sub-maximum level (90%VO_2max_). The study showed that although the change in exercise performance of the subjects was not significant, it appears that acute inspiratory muscle preparation activity may cause some changes in the cardiopulmonary system by changing the breathing rate, mainly manifested by a decrease in heart rate and an increase in the cardiopulmonary system efficiency. A review of published studies on IMW shows that the intensity of acute IMW intervention is generally designed to be 40% MIP, while the initial intensity of long-term respiratory muscle training is 40% ~ 50% MIP, and increases at a rate of about 5% considering the athlete’s tolerance to the intervention.

When the inspiratory muscle is weak or fatigued, its blood flow will decrease compared to normal conditions, but the proportion of respiratory muscle blood flow to cardiac output during exercise can increase from 2% to 16% in normal people [15],Without enough blood perfusion, the body’s ability to move will be greatly reduced. In addition, with the change of body posture, the length-tension relationship of the diaphragm will also change, which will lead to the decrease of breathing depth and breathing economy of athletes. Compared with upright running posture, training in sitting posture such as bicycle riding and rowing may reduce the abdominal cavity space, and reduce the chest lifting and chest and abdominal movement, which limits the contraction and relaxation of the diaphragm. Maintaining this state for a long time will cause the weakening of diaphragm function, leading to excessive participation of intercostal muscles around the chest and fatigue of the inspiratory muscles. This also suggests the need for IMW interventions for rowers prior to training.

Combined with the above results, the study concluded that under the condition that the respiratory muscle is in poor condition or not activated, its working efficiency is also low, the blood flow allocated by the body to the inspiratory muscle will increase and the corresponding blood flow of the motor muscle will decrease, and the phenomenon of the inspiratory muscle stealing the blood flow of the motor muscle is more likely to occur, resulting in insufficient energy supply to the motor muscle and a decrease in exercise endurance. This should be avoided as far as possible in rowing endurance training. As a specific respiratory muscle warm-up program, IMW can improve the function of the inspiratory muscle, relieve the perception of dyspnea and reduce the work done by the respiratory muscle, thus affecting the oxygen supply of the exercising muscle during constant power training. In the long term, the quality of ergometer training will be improved, allowing athletes to achieve better aerobic endurance reserve and ultimately improved athletic performance.

### 4.2. Effect of IMW intervention on brain activation level

In this study, changes in brain activation levels were analyzed from two perspectives: the CR10 scale, representing subjective perceived exertion, and PFC oxygenation status, was verified from an objective perspective. Based on prefrontal cortex anatomy, the ventral prefrontal cortex was further divided into the ventromedial prefrontal cortex (VMPFC) and the orbitofrontal cortex (OFC). The VMPFC is closely connected to the limbic system and autonomic system, while the OFC is more connected to the sensory system. The difference in the oxygenation level of the PFC region in different groups may indicate that the IMW intervention weakened the feedback of the cardiopulmonary system, and reduced the discomfort of the subjects, which was manifested as the reduction of CR10 level. However, since the study was carried out in the actual training activities, no more channels of fNIRS equipment were used to collect whole brain indicators, and this result needs to be confirmed by further experiments. Taken together, we suggest that during ergometer training, afferent feedback from peripheral tissue systems may be transmitted to regions surrounding the PFC, and IMW intervention may have a positive effect on these psychophysiological changes.

On whether the PFC is an effective area for monitoring physical perception, Brass designed an experiment that used signals to control subjects to perform/suppress keystrokes, and evaluated subjects’ brain activation characteristics through MRI under different conditions. He found that a small area near bilateral AIC and MPFC is specifically involved in active, volitional inhibition of motor behavior [16];In the study of the right PFC region, Fares A [17] used electroencephalogram (EEG) technology to monitor EEG signals in the prefrontal region to judge physiological changes of subjects, and used Nasa task load index and NASA-TLX questionnaire to measure stress and its effects. This study identified PFC regions in relation to psychological stress levels, making clear the relationship between stress tasks and motor performance as well as commonly used scales. In general, the incoming feedback from the thalamic system goes through some complex calculations to reach the OFC and VMPFC regions and is integrated with sensory information [18]. The results of electrophysiological and magnetic resonance imaging (MRI) experiments also show that the above two brain areas are important in executive control and reward sensitivity [19], emotional state [20], value estimation [18], and will be activated when inhibiting negative emotions. Based on the particularity of OFC activity and its functional connectivity, this area may be the key brain area for value judgment and emotion. It plays an important role in assessing value and maintaining emotional states and ultimately making behavioral decisions. The effect of IMW intervention was further supported by the reduction in the overall activation level in the PFC region.

According to Wickens’ research [21] on cognitive load and the kuznets curve relationship between mental load and sports performance, it can be seen that rowers may mobilize additional mental resources to maintain the established power output during the continuous training process, which is manifested as the difference in the activation level of the frontal lobe between the control group and the experimental group. If the activation level of the frontal lobe is further deepened, The compensation mechanism of fatigue may occupy more cognitive resources and reduce the information processing ability of athletes [22,23],On the one hand, it will affect the normal progress of athletes’ training. On the other hand, although the training intensity is unchanged or will continue to increase, neither the cognitive load nor the mental resources of athletes will change significantly. This is because their brain resource capacity is limited and they are unable to allocate more neural resources to meet the requirements of training. Therefore, the probability of the athlete’s technical movement irregularity will be increased. If no intervention is applied or the athlete is forced to maintain the set power output as much as possible, over time, it will not only have a negative impact on the athlete’s sports performance, but also may cause sports injury.

At present, there are many empirical studies on the recovery measures after high intensity in the published literature [24–27],1,2,However, how to regulate the subjective load before or during training and promote athletes to complete the same or even higher load training plan with lower physical perception is one of the problems to be solved. The training load of rowing is extremely high, which stimulates the heart, lung and muscle system. At the same time, this sport also puts forward higher requirements for the special technology of athletes. In order to achieve the required physical fitness and technical level of the competition, no matter the elite athletes or the athletes who have just started the training of the cross-event selection, they will carry out more ergometer training. As a training method similar to the special technical movements of rowing, ergometer training is not affected by hydrological and environmental factors, so it has been favored by more coaches, but for athletes, the total load of ergometer training is higher, and the muscles and cardiopulmonary system have strong discomfort during training. Therefore, in actual training, athletes often cannot meet the load intensity prescribed by coaches. At this time, coaches often adopt some incentive or punishment measures to promote them to complete the training plan, which makes the subjective load of athletes continue to maintain at a high level, and the neural resources are limited [28], If this state lasts too long, it will affect their information processing ability, the ability of central recruitment of neurons will also be reduced, and it will be more difficult to meet the requirements of training. The regulation of perceived exertion may promote them to better meet the requirements and goals of the training plan. Future studies should enhance monitoring accuracy by employing high-density fNIRS arrays (e.g., 128-channel systems with 20–30 mm optode spacing) to expand cortical coverage while improving spatial resolution. Integrating EEG-fNIRS multimodal recordings would capture complementary neural dynamics across hemodynamic and electrophysiological timescales. Advanced artifact correction combining inertial motion sensors and deep learning algorithms could mitigate exercise-induced signal noise. Such a framework would enable precise identification of neural biomarkers governing subjective load formation through spatiotemporal refinement of whole-brain monitoring.

Interventions such as surface neuromuscular electrical stimulation [29] and transcranial direct current stimulation [30], that can reduce subjective load and improve performance will be applied more scientifically in the preparation activities or recovery of athletes. To provide more feasible strategies for improving the efficiency and quality of athletes’ training.

### Limitations

Our study has several limitations. First, the PCA-based motion artifact correction in statistical processing may inadvertently remove meaningful physiological data, potentially affecting the interpretation of HbO_2_ changes. Second, the experimental instruments used might have contributed to non-significant findings due to technical constraints. Although we employed randomized grouping and statistically adjusted athletes’ resting-state prefrontal HbO_2_ as baseline values to minimize confounding effects, unmeasured factors such as genetic predispositions and stress levels could introduce residual variability. Future research should incorporate multimodal assessments (e.g., functional MRI, genetic analyses) to better elucidate these underlying sources. In addition, load control is helpful to maintain moderate chronic load, promote active adaptation, improve competitive ability and reduce injury rate. The study presents an IMW method based on previous intervention schemes and analyses the acute effect of IMW. In order to achieve the objective of optimizing the quality of training and improving athletes’ performance, it is necessary to conduct long-term research on interventions for the respiratory muscles.

## 5. Conclusion

By systematically monitoring athletes’ perceived exertion, muscle oxygen saturation and brain activation characteristics, this study demonstrated the effectiveness of IMW in reducing perceived exertion and optimizing muscle oxygenation. These findings suggest that coaches and sports practitioners may consider incorporating IMW into standardized training regimens to enhance endurance training quality. Future research should elucidate the longitudinal effects of IMW on athletes’ perceived exertion and tissue oxygenation kinetics, particularly in relation to training adaptation and performance outcomes.

## Supporting information

S1 Data set Complete dataset of athlete performance metrics from lab and field tests. (XLSX)
